# Segmentation of Heart Sound Signal Based on Multi-Scale Feature Fusion and Multi-Classification of Congenital Heart Disease

**DOI:** 10.3390/bioengineering11090876

**Published:** 2024-08-29

**Authors:** Yuan Zeng, Mingzhe Li, Zhaoming He, Ling Zhou

**Affiliations:** 1 Research Center of Fluid Machinery Engineering and Technology, Jiangsu University, Zhenjiang 212013, China; 2212111005@stmail.ujs.edu.cn (Y.Z.); 2212211038@stmail.ujs.edu.cn (M.L.); hezhaoming@ujs.edu.cn (Z.H.); 2 Department of Mechanical Engineering, Texas Tech University, Lubbock, TX 79411, USA

**Keywords:** congenital heart disease, heart sound segmentation, multi-scale feature fusion, multi-classification

## Abstract

Analyzing heart sound signals presents a novel approach for early diagnosis of pediatric congenital heart disease. The existing segmentation algorithms have limitations in accurately distinguishing the first (S1) and second (S2) heart sounds, limiting the diagnostic utility of cardiac cycle data for pediatric pathology assessment. This study proposes a time bidirectional long short-term memory network (TBLSTM) based on multi-scale analysis to segment pediatric heart sound signals according to different cardiac cycles. Mel frequency cepstral coefficients and dynamic characteristics of the heart sound fragments were extracted and input into random forest for multi-classification of congenital heart disease. The segmentation model achieved an overall F1 score of 94.15% on the verification set, with specific F1 scores of 90.25% for S1 and 86.04% for S2. In a situation where the number of cardiac cycles in the heart sound fragments was set to six, the results for multi-classification achieved stabilization. The performance metrics for this configuration were as follows: accuracy of 94.43%, sensitivity of 95.58%, and an F1 score of 94.51%. Furthermore, the segmentation model demonstrates robustness in accurately segmenting pediatric heart sound signals across different heart rates and in the presence of noise. Notably, the number of cardiac cycles in heart sound fragments directly impacts the multi-classification of these heart sound signals.

## 1. Introduction

To ensure improved treatment outcomes, there is need for early recognition, convenient diagnosis, and treatment of congenital heart diseases The acoustic characteristics of the heart sound signal directly reflect the mechanical activity of the heart, serving as a crucial foundation for assessing cardiac pumping function. However, individual physician factors in terms of accuracy and efficiency influence traditional diagnostic techniques such as stethoscopes and echocardiography, including differences in auditory perception, professional skill level, and clinical experience [1,2,3]. Therefore, it is important to develop an objective and efficient method for the automatic analysis of heart sound signals to aid in the prevention and clinical management of congenital heart disease.

**Figure 1:**
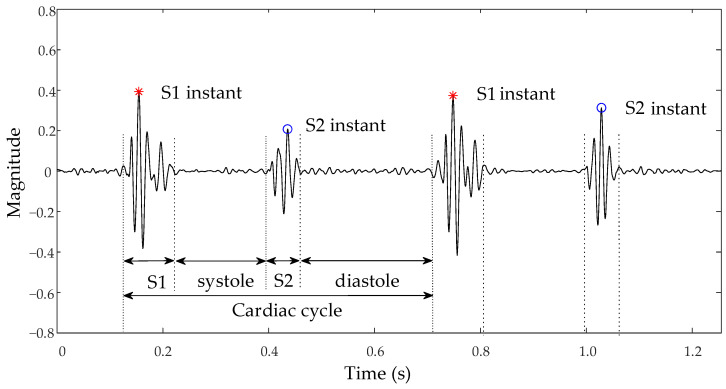
Schematic of normal heart sound signal and nomenclature.

Analysis of the heart sound signal, particularly the first (S1) and second (S2) heart sounds, is crucial for precisely identifying ventricular contraction and relaxation initiation points to evaluate cardiac pumping efficiency [4]. Abnormal murmurs in heart sounds, such as additional S3 and S4 sounds or pathological murmurs, not only indicate the presence of heart disease but also reveal specific sites of pathology, such as valve stenosis or insufficiency [5]. For instance, the heart sound characteristics of an atrial septal defect (ASD) may feature a split S1, due to the increased filling of the right ventricle, which leads to delayed tricuspid valve closure. Furthermore, if pulmonary artery hypertension is present, the S2 may be accentuated or split [6]. On the other hand, the heart sound characteristics of a ventricular septal defect (VSD) may include a harsh systolic murmur, typically heard at the left sternal border between the third and fourth intercostal spaces, possibly accompanied by a systolic thrill. If there is a significant shunt, a functional diastolic murmur may appear at the cardiac apex [7]. Careful analysis of heart sound characteristics allows for further disease severity assessment and provides critical information for clinical decision-making. Therefore, accurately segmenting the heart sound signal to locate fundamental heart sounds is a vital step in automatic analysis. The nomenclature for each state of the heart sound signal is presented in Figure 1. In the cardiac auscultation cycle, the period from the end of S1 to the beginning of S2 corresponds to the Systole, and the interval from the end of S2 to the start of the next S1 represents the Diastole.

In previous studies, heart sound segmentation methods have been broadly classified into peak selection algorithms, statistical model-based methods, and deep learning [8]. The peak selection algorithm involves obtaining the envelope of the heart sound signal and differentiating S1 and S2 peaks using methods such as wavelet transform [9], Hilbert transform [9], and variational mode decomposition [10], among others. The accuracy of the algorithm is influenced by the relationship between the duration of systolic and diastolic periods. The segmentation methods based on statistical models [11,12,13,14] require the utilization of a heart sound activity detection algorithm to estimate the probability of each frame signal corresponding to each heart sound event. These probabilities are subsequently fed into the probability model and used to deduce the optimal state sequence through decoding algorithms like the Viterbi algorithm, thereby automatically segmenting heart sound events [15,16]. The statistical model-driven location segmentation technique demonstrates validity in noisy environments, but its performance is heavily reliant on the accuracy of the heart sound detection algorithm. In the segmentation of heart sounds, the convolutional neural network [4,17] or recurrent neural network [18,19,20] is trained using time-frequency domain features extracted from heart sound signals to achieve automatic segmentation. However, the segmentation of heart sound signals still faces huge challenges due to non-stationarity, individual differences, and susceptibility to various noises [21].

The classification of heart sounds involves a structured process primarily focusing on feature extraction and classifier design [22], with the features mainly consisting of time domain [23] and frequency domain features [24]. The multi-scale feature extraction method facilitates a more comprehensive analysis of signal characteristics by examining the signal across various time and frequency scales [25,26]. The classifiers used include support vector machines [27,28], random forests [29], and neural networks [30,31]. ASD and VSD are the most common types of congenital heart disease [32], and their accurate classification, along with that of normal heart sounds, is essential for early detection and management. However, the investigation into the multi-classification of congenital heart disease remains limited, with existing studies predominantly reliant on researcher-constructed datasets. Specifically, existing studies have issues such as not focusing on the multi-classification of congenital heart disease [33], the proposed models having a low classification accuracy rate for ASD [34], and the constraints of a small sample size [35]. Furthermore, detailed exploration of how the number of cardiac cycles within heart sound signals impacts classification performance has not been thoroughly addressed in current literature.

Although numerous algorithms are available for automatically classifying heart sound signals, most current studies focus on analyzing heart sound fragments of equal duration [36,37]. However, there is still insufficient information about the significance of cardiac cycle analysis in distinguishing pathologies. Moreover, the challenge of the multi-classification of heart sound signals is persistent because of inadequate datasets. This study proposes the TBLSTM algorithm, which is based on an enhanced temporal convolutional network (TCN) and a bidirectional long short-term memory network (Bi-LSTM), to identify each state in children’s heart sound signals accurately. The study also examines the impact of heart sound fragments with different cardiac cycles on the multi-classification task of congenital heart anomaly.

## 2. Materials and Methods

### 2.1. Datasets

In the absence of manual annotations, the electrocardiogram (ECG) offers a precise temporal reference for heart sounds. Specifically, S1 typically coincides with the R-wave of the ECG, indicating the onset of ventricular systole, whereas S2 corresponds to the conclusion of the T-wave, denoting the transition to diastole [38,39]. The dataset utilized in this study was collected from the Massachusetts Institute of Technology Heart Sounds Database (MITHSDB). The study employs both the integrated and filtered versions of the MITHSDB as training and test sets. The dataset consisted of synchronized heart sounds and ECG signals, from which samples affected by extreme noise or unclear synchronous ECG signals were systematically excluded [21]. The final dataset comprised 576 heart sound samples, each lasting 5 s and down-sampled to 2 kHz. In addition, a separate dataset from a self-collected experimental group served as the test set to evaluate the performance of the segmentation model. This dataset was also utilized for conducting a multi-classification study focused on congenital heart diseases [9]. The stethoscope utilized in data collection employed a piezoelectric pickup method with a hardware sampling rate of 44.1 kHz and a band-pass range of 30–500 Hz through three pickup modes for 16-bit AD sampling, and the data were stored in .wav format. The dataset encompassed heart sounds from 98 healthy children, 55 patients with ASD, and 68 patients with VSD in various clinical settings and regions that had various types of noise. Notably, the ASD cases often presented with tricuspid regurgitation, while the VSD cases exhibited specific cardiac structural abnormalities.

### 2.2. Normalization and Denoising

A standardized pre-processing procedure was executed on all the heart sound samples to ensure uniformity and comparability. The second-order Butterworth bandpass filter with a frequency range of 25 to 400 Hz was applied for noise reduction, followed by down-sampling the signal to 2 kHz and applying the maximum normalization method for signal normalization.

### 2.3. Segmentation Method

#### 2.3.1. Envelope Extraction

Heart sound signals contain valuable characteristic information in the time-frequency domain. This study extracted four heart sound signal envelopes as segmentation features, namely the Hilbert envelope, homomorphic envelope, wavelet envelope, and power spectral density envelope. The Hilbert envelope was identified as the absolute value of the Hilbert transformation of the heart sound signal. The homomorphic envelope was obtained by applying a zero-phase low-pass Butterworth filter to extract the logarithm of the absolute value after performing a Hilbert transformation on the original signal. The wavelet envelope was obtained through discrete wavelet transform decomposition of the heart sound signal using Daubechies6 as the parent wavelet [24], extracting high-frequency detail coefficients and low-frequency approximate coefficients using the high pass and low pass filters respectively, with the wavelet envelope being defined as the absolute value of third layer high-frequency detail coefficients. The power spectral density envelope represents the mean power spectral density over a specific frequency range [40]. Hamming Windows with a window size of 0.05 s and 50% overlap were used for segmenting signals, obtaining their frequency domains by windowing and short-time Fourier transform. The term power spectral density refers to the square amplitude when analyzing frequency domain analysis.

#### 2.3.2. TBLSTM

**Figure 2:**
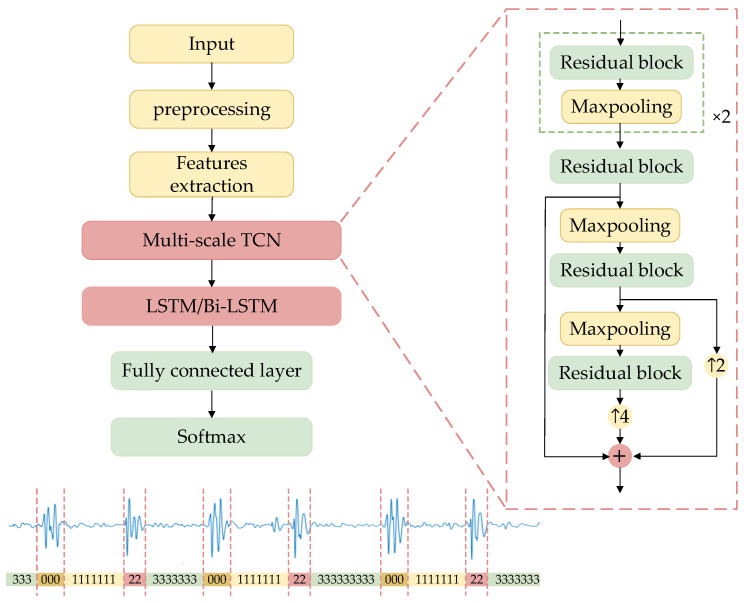
The structure of TLSTM and TBLSTM.

Figure 2 clearly illustrates the architecture of the segmentation network. Time-frequency domain features of heart sound signals were used to train the segmentation model, which integrated a multi-scale TCN with an LSTM or a Bi-LSTM to discern the state of heart sounds accurately at each moment. The multi-scale TCN was structured as a sequence of alternating residual blocks and max-pooling layers, with upsampling indicated by arrows, and the numerical values alongside indicating the respective upsampling rates. Initially, the algorithm extracted multi-scale time-frequency features of the signal using the enhanced TCN and subsequently captured the long-term dependencies and dynamic features of the signal through the LSTM or Bi-LSTM layers. Dropout technology was introduced after the Bi-LSTM layer for random data deactivation processing to enhance the model’s generalization capability and mitigate overfitting risks. The integration of fully connected layers with the Softmax function translated high-dimensional feature mappings into probabilities for each class, thus generating labels corresponding to four states of heart sound signals: 0 for S1, 1 for systole, 2 for S2, and 3 for diastole.

**Figure 3:**
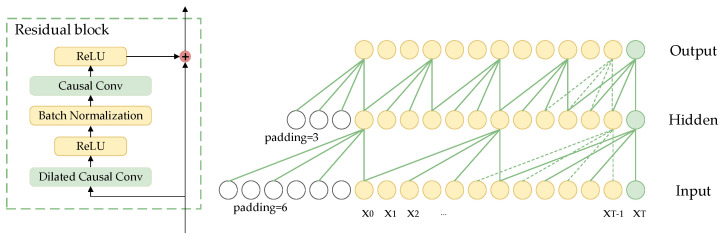
Structure diagram of residual block in TCN.

The TCN is a deep learning method based on the structure of convolutional neural networks [41]. The TCN network served as the fundamental architecture for multi-scale feature fusion, with its primary components being stacked residual blocks that formed the main framework of TCN. Figure 3 clearly illustrates the specific design details for every residual block. Each residual block is made up of a dilated causal convolutional layer, a causal convolutional layer, and a rectified linear unit (ReLU) activation function. Causal convolution ensures that the model only uses information from the current and past moments to generate output at each moment, without accessing any future information. The dilated convolution expands the receptive field by introducing a specified number of zeros between the elements of the convolution kernel according to the dilation rate, allowing for skipping parts of the input and expanding the receptive field to apply the kernel over a larger region.

The input to the TCN network is the heart sound features X(n). The convolution kernel of each layer is represented by F(k). The calculation formulas for causal convolution and dilated causal convolution at $x_{n}$ are given by (1) and (2), respectively.

(1) $${\left(F*X\right)}_{\left(x_{n}\right)}=\sum_{k=1}^{K}f_{k}x_{n-K+k}$$

(2) $${\left(F{*}_{d}X\right)}_{\left(x_{n}\right)}=\sum_{k=1}^{K}f_{k}x_{n-\left(K-k\right)d}$$

where the symbol ∗ denotes the causal convolution and the symbol ${*}_{d}$ denotes dilated causal convolution, K represents the quantity of convolution kernel, and d represents the dilation rate. The dilation rate for dilated causal convolution in the five residual blocks is sequentially 2, 4, 8, 16, and 16.

The dilated causal convolution receptive field has a size of (K−1)×d+1, and to maintain consistent input and output sequence lengths, each layer requires zero-padding on the left side of the input sequence by a length of (k−1)×d.

Due to its simple structure, a single residual block struggles to capture complex and multi-level features in the signal. Although the straightforward series of residual blocks mitigates the issue of gradient disappearance through skip connections, it remains inadequate for addressing the multi-scale characteristics of heart sound signals, thereby constraining the model’s capability in detailed feature extraction. Pathological features in heart sound signals tend to appear at different time scales; hence, introducing a maximum pooling layer between the residual blocks facilitates multi-scale feature learning. The output of each residual block is down-sampled using a maximum pooling layer with a window size and sliding step size both set at 2. Employing a sliding window approach to select the maximum output value within each window effectively reduces feature length while retaining crucial change points within the feature. Different residual block outputs are standardized through interpolation, integrating abstract features from various levels while maintaining spatial resolution. Ultimately, this process yields multi-scale time-frequency features of heart sound signals. The multi-scale fusion method enables the extraction of feature information from signals with varying resolutions and enhances the sensitivity of TCN to local dependencies within sequential data.

**Figure 4:**
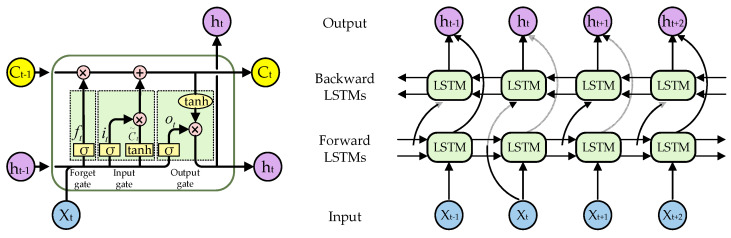
The structure of the LSTM unit and Bi-LSTM.

The LSTM is a type of vanilla recurrent neural network, and the Bi-LSTM is a specialized variant of the LSTM, as depicted in Figure 4. The Bi-LSTM addresses the limitations of the vanilla LSTM model by incorporating feature merging extraction steps for both forward and reverse sequences, enabling it to effectively capture contextual information from past and future data points. The LSTM network is composed of a sequence of repeated LSTM units, with each unit containing three gated structures—a forget gate, an input gate, and an output gate, as well as a cell state. Through its gating mechanism, the LSTM can regulate information flow by selectively retaining or discarding past information which subsequently influences the content of the present output.

(3) $$\left\{ \begin{array}{l} i_{t}=\sigma \left(W_{i}\left[h_{t-1},x_{t}\right]+b_{i}\right)\\ f_{t}=\sigma \left(W_{f}\left[h_{t-1},x_{t}\right]+b_{f}\right)\\ o_{t}=\sigma \left(W_{o}\left[h_{t-1},x_{t}\right]+b_{o}\right)\\ {\tilde{C}}_{t}=\tanh\left(W_{C}\left[h_{t-1},x_{t}\right]+b_{C}\right)\\ C_{t}=f_{t}*C_{t-1}+i_{t}*{\tilde{C}}_{t}\\ h_{t}=o_{t}*\tanh\left(C_{t}\right) \end{array}\right.$$

where i, f, o and C denote input gate, forget gate, output gate, and cell memory, respectively. W and b are the weight and bias terms, respectively. $x_{t}$ and $h_{t}$ denote the input and output of LSTM at step t. In addition, σ denotes sigmoid activation and ∗ denotes dot product.

The TBLSTM model was utilized to identify each state of the heart sound signal in a pediatric heart sound dataset. The Adam optimizer was utilized for the training process with a learning rate set to 0.001, and Cross Entropy was chosen as the loss function. The batch size was configured to 32 and the number of training rounds was set to 90.

A sequential segmentation strategy was implemented to progressively increase the length of the cardiac cycle. The initial segmentation yielded a fragment of a single cardiac cycle, and each subsequent segmentation added another cardiac cycle while maintaining overlap with adjacent fragments. This approach not only mimics the natural continuity of the heart sound signal but also systematically evaluates the adaptability and generalization ability of the heart sound processing model to changes in the cardiac cycle by incrementally increasing the number of included cycles.

### 2.4. Features Extraction and Classification

In the field of heart sound signal processing, the Meir frequency cepstral coefficient (MFCC) is a widely utilized technique for feature extraction. By simulating the auditory perception mechanism of the human ear, MFCC effectively captures the acoustic characteristics of sound signals. In this present study, the heart sound signal underwent preprocessing to reduce noise levels and was then divided into short time frames with a duration of 20 ms and a frameshift of 10 ms. These frames were subsequently transformed into the frequency domain using a fast Fourier transform (FFT). Within the frequency domain, a Meir frequency filter bank with cutoff frequencies at 30 Hz and 500 Hz, respectively, consisting of 26 filters, processed the signal to obtain energy distribution on the Meir frequency scale. The logarithmic energy was then converted to MFCC through discrete cosine transform (DCT). The order of extracted MFCC was set at 12, and its derivative features were obtained by extracting first-order and second-order time-derivatives in MFCC features. This study focused on extracting MFCC and its derivative features with a total feature dimensionality of 39 dimensions—including 13 dimensions each for MFCC itself, its first-order time-derivative, and its second-order time-derivative. The MFCC features, reduced by principal component analysis and subsequently normalized, were fed into the random forest classifier to conduct multi-classification on heart sound signals, and the random forest model consisted of 100 trees.

### 2.5. Evaluation Metric

The concept of the tolerance time window was introduced to quantitatively evaluate the performance of the heart sound signal segmentation model, accounting for the difference in duration between S1 and S2 in different heart sound samples. To achieve this, small deviations in segmentation results were noted by setting a time threshold. Specifically, the tolerance time windows for S1 and S2 were set at 100 ms and 80 ms respectively. In the task of heart sound segmentation, a predicted heart sound state falling within the tolerance time window of the true heart sound state was considered as true positive (TP) or true negative (TN). Any other incorrectly predicted heart sound states within this tolerance time window were considered false positive (FP) or false negative (FN). The evaluation metrics utilized included accuracy, sensitivity, precision, and F1 score, which are defined as (4):

(4) $$\begin{array}{cc} Acc= & \frac{\mathrm{TP}+\mathrm{TN}}{\mathrm{TP}+\mathrm{FP}+\mathrm{TN}+\mathrm{FN}}\\ Se= & \frac{\mathrm{TP}}{\mathrm{TP}+\mathrm{FN}}\\ P= & \frac{\mathrm{TN}}{\mathrm{TN}+\mathrm{FP}}\\ F_{1}= & \frac{2\times Se\times P}{Se+P} \end{array}$$

The macro average method was employed in this paper to combine the overall sensitivity and accuracy, considering the presence of four different states within the heart sound signal.

In the process of evaluating multi-classification performance, the same evaluation metrics employed during the segmentation stage were used. This approach was taken due to an equitable distribution of samples across all categories (1200:1200:1200), thereby resulting in macro average accuracy aligning with overall accuracy. As a result, only the accuracy index was displayed in the table.

## 3. Results

### 3.1. Segmentation Results

Table 1 compares the performance of the heart sound signal segmentation model proposed in this study with existing segmentation models. Additionally, the Feature Pyramid Network (FPN) model [42], widely utilized in the field of visual segmentation, was introduced for comparative analysis. All models in this study were experimentally reproduced based on the methodologies described in the original literature. To ensure fairness and consistency, all models were tested on a standardized dataset with uniform pre-processing steps, feature extraction methods, and evaluation criteria. Furthermore, to validate algorithm robustness, the experiment employed a six-fold cross-validation, with all values in the table representing average results from this method.

The TBLSTM model demonstrated superior performance across all evaluation metrics, except sensitivity. The overall accuracy and F1 scores of the model reached an impressive 94.15%. While the TBLSTM model achieved optimal F1 scores for both first and second heart sounds compared to other models, its F1 scores for these specific heart sounds were only 90.25% and 86.04%, respectively.

**Figure 5:**
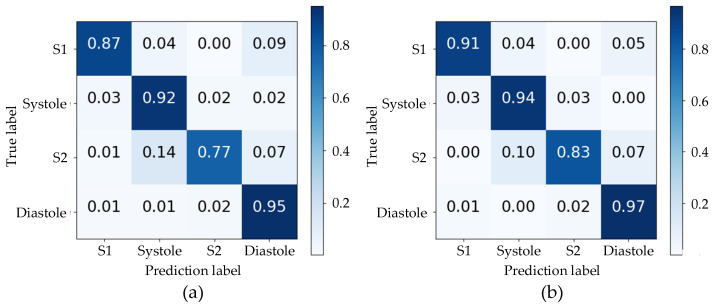
The confusion matrixes for the segmentation results of two models: (**a**) the TLSTM model; (**b**) the TBLSTM model.

The accuracy of the proposed algorithm’s segmentation was evaluated using the normalized confusion matrix in Figure 5 on the test set. The use of envelope features and a TBLSTM model resulted in notably high prediction accuracy for all states with values of 91%, 94%, 83%, and 97% respectively. A comparative analysis between (a) and (b) in Figure 5 reveals that the TBLSTM model exhibits a notable enhancement in segmentation accuracy compared to TLSTM, particularly evident in its improved ability to accurately identify S1 and S2. This is characterized by the model’s capacity to label these segments with greater precision. Notably, among all algorithms, the lowest prediction accuracy was observed for S2, primarily due to misjudgments as Systole with a probability exceeding 10%. This may be attributed to weak sound characteristics of S2 and unclear time boundaries with Systole and Diastole resulting in predictions into other states. Despite similar durations of Systole and Diastole intervals in the cardiac cycle, their accurate prediction by the TBLSTM model reached 94% and 97% respectively through learning complex time series features of heart sounds.

**Figure 6:**
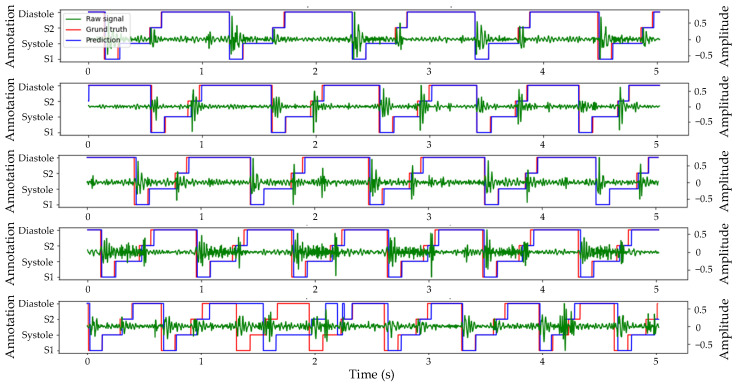
Example of prediction and ground truth of heart sound segmentation based on envelopes and TBLSTM.

Figure 6 displays the Prediction, Ground truth, and corresponding Raw heart sound signal in the test set. The predicted results are indicated in blue, the original annotation in red, and the original heart sound signal in green. All displayed heart sound signals had a duration of 5 s. The first example exhibited the highest segmentation accuracy in the test set with four cardiac cycles and a slight murmur. The predicted output of the algorithm was highly consistent with the real label with only a minor deviation. In the second and third examples, despite variable durations of systolic and diastolic periods within five cardiac cycles, the algorithm accurately identified each state of the cardiac cycle with only slight prediction deviation. Even amidst discernible noise during systole in the fourth example, six cardiac cycles within 5 s were accurately identified by the algorithm. However, in the last examples, due to low and close amplitudes of S1 and S2, and pronounced high-amplitude murmurs during the fourth cardiac cycle, the algorithm began to misjudge from the second cycle, with the most severe misjudgment occurring in the fourth cycle.

**Figure 7:**
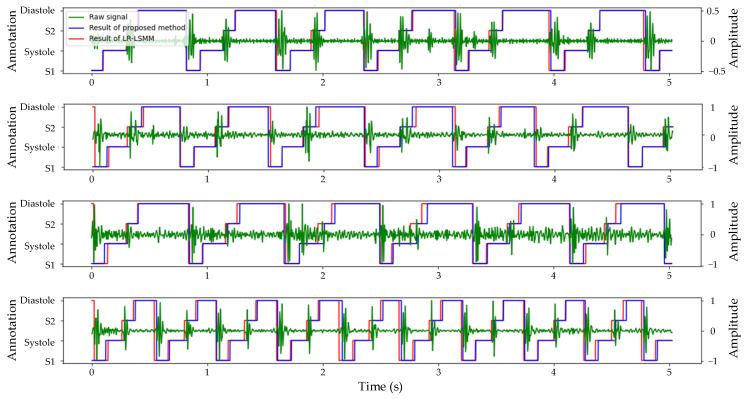
Comparison of the segmentation results of TBLSTM and LR-HSMM for the same heart sound signal.

The segmentation model exhibited exceptional performance on the validation set. To further evaluate the model’s generalization capability, we utilized the heart sound dataset of the experimental group as the test set, and the segmentation results are illustrated in Figure 7. Since corresponding ECG signals were not available, conducting a quantitative analysis of segmentation effectiveness was unfeasible. However, it is evident from the evaluation metrics in Table 1 that both the logistic regression and hidden Semi-Markov model (LR-HSMM) algorithms ranked second best across all evaluation indicators compared to the algorithm proposed in this study. To validate the segmentation performance of our proposed algorithm, we applied the LR-HSMM algorithm to segment the same heart sound signal and compared its results with those obtained using our proposed algorithm.

Considering that the majority of patients with ASD and VSD were accompanied by tricuspid valve regurgitation (TR) symptoms, this study further extended its analysis to focus on the heart sound signals specifically indicative of tricuspid valve regurgitation symptoms. The segmentation results for normal heart sounds, ASD, and VSD are displayed in Figure 7. The segmentation results of different heart sound signals are presented in Figure 7. The first line illustrates the segmentation of normal heart sound signals, where the presence of noise has no significant impact on the accuracy of segmentation. The subsequent examples depict the segmentation of ASD and VSD heart sound signals. Despite the potential challenges such as the obscured S1 in ASD and a whole murmur in VSD, both the proposed segmentation algorithm in this study and the LR-HSMM algorithm can accurately identify the location of S1 and S2 heart sounds. Lastly, the final example illustrates the segmentation results of a heart sound signal with TR symptoms only, showing a significant increase in cardiac cycles without compromising accurate localization by both algorithms.

### 3.2. Classification Results

**Figure 8:**
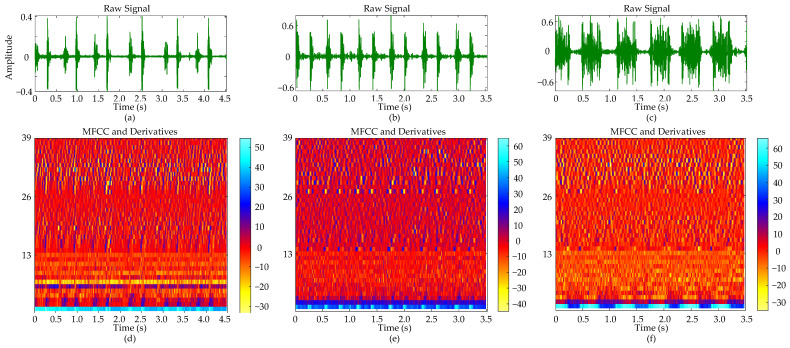
Comparative analysis of heart sound signals and their MFCC representations: (**a**) Normal heart sound signal with six cycles; (**b**) ASD heart sound signal with six cycles; (**c**) VSD heart sound signal with six cycles; (**d**) MFCC and its derivatives of the normal heart sound signal; (**e**) MFCC and its derivatives of the ASD heart sound signal; (**f**) MFCC and its derivatives of the VSD heart sound signal.

Figure 8 presents a comparative analysis of cardiac auscultation signals from three distinct cases: a normal subject, a patient with ASD accompanied by tricuspid regurgitation, and a patient with VSD characterized by a 0.66 cm defect located beneath the aortic valve, right sinus downward prolapse, and partial obstruction of the ventricular septal defect, along with mild mitral and tricuspid regurgitation. In the raw cardiac sound signals (a–c), the normal subject exhibits S1 with lower amplitude compared to S2; the ASD patient shows a more pronounced S1; and the VSD patient presents S1 and S2 with comparable amplitudes. The duration of six cardiac cycles is approximately 4.50 s for the normal subject and about 3.50 s for both ASD and VSD patients.

The MFCC and its derivatives (d–f) provide a spectral analysis that complements the observations obtained from time-domain analysis. The color bar within the figure provides a visual mapping of the magnitude of the MFCC and its derivatives. Regions with higher values typically correspond to parts of the signal where energy is more concentrated or spectral features are more pronounced. The MFCC coefficients for the normal cardiac sound (d) display a relatively uniform color distribution across the time axis, indicating a stable spectral envelope. For the ASD patient (e), the MFCC image shows distinct S1 and S2, while in the VSD patient (f), an increase in color intensity within specific time frames corresponds to energy concentration in the systolic murmur regions. The first and second derivatives exhibit more dynamic color changes compared to the MFCC, revealing rapid spectral variations over time.

In the multi-classification of heart sound signals, we employed the six-fold cross-validation method to evaluate the model’s performance on the dataset. The table displays the average evaluation metrics.

**Figure 9:**
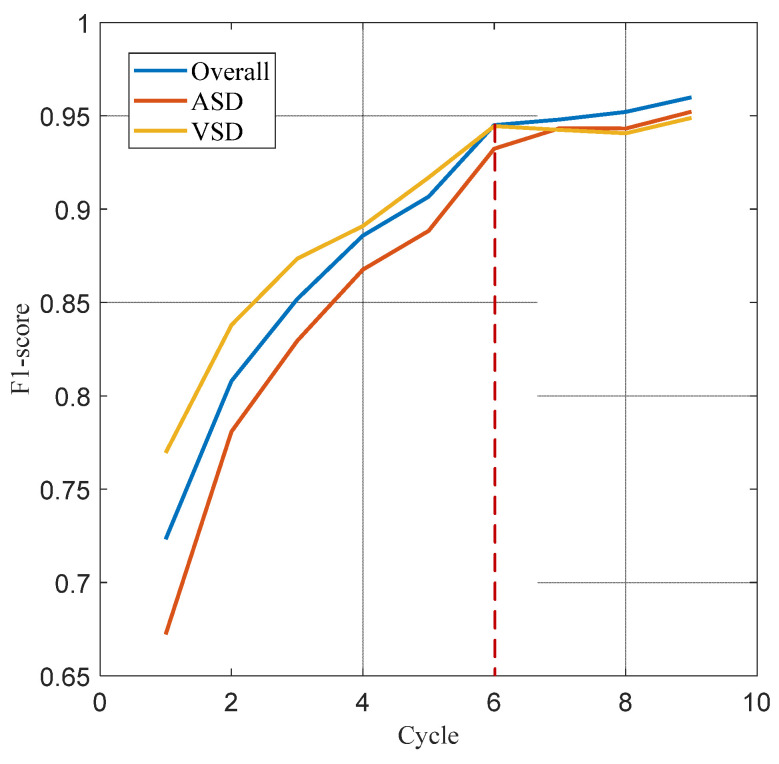
F1 score curves for the overall, ASD, and VSD.

The data presented in Table 2 demonstrates the impact of different cardiac cycles on the performance of the heart sound signal segmentation model. It is evident that as the number of cardiac cycles increases, all performance indicators exhibit a consistent upward trend until they stabilize after six cardiac cycles. The model demonstrated stabilization in performance after six cardiac cycles, with sensitivity, accuracy, and F1 scores achieving 94.43%, 95.58%, and 94.51%, respectively. When the number of cycles was fewer than six, the variations between adjacent cycle counts for various indicators exceeded 1%. Conversely, when the number of cycles was six or more, these variations were contained within 1%. Figure 9 further indicates that the number of cardiac cycles also positively influences the classification of ASD and VSD, with F1 scores reflecting variations in classification performance among pathological types. The F1 scores for ASD pathologic types were consistently lower than the overall F1 scores, while those for VSD types were higher than the overall F1 scores before six cardiac cycles but fell below it afterward.

## 4. Discussion

This article describes the use of a fusion model comprising a convolutional neural network and recurrent neural network for heart sound segmentation, leveraging both the short burst characteristics within a cardiac cycle and the temporal dependencies across cardiac cycles. Our proposed model integrates a multi-scale time convolutional network and a bidirectional long short-term memory network to accurately segment heart sound signals. The multi-scale TCN architecture effectively captured the characteristics of heart sound signals on different time scales, while the bidirectional information flow characteristics of Bi-LSTM significantly enhanced the recognition ability of signal rhythm and periodic characteristics. The combination of these two enhanced the performance of heart sound signal segmentation, enabling multi-classification of heart sound signals.

### 4.1. Segmentation

This study preliminarily evaluated the segmentation results of both the proposed model and existing models. In the recognition of S1 and S2 heart sounds, none of the models achieved an F1 score that exceeded the overall average level, with S1 generally outperforming S2. This discrepancy in the evaluation scores would be attributed to several factors, including the similarity between S1 and S2, the unpredictability of systolic duration, and the presence of high-amplitude and persistent murmurs, all of which affected the models’ consistency in segmenting S1 and S2. Furthermore, the position and characteristics of S1 remain relatively stable across cycles, while the timing of S2 within the cardiac cycle may vary with changes in heart rate, leading to variability in their occurrence [43]. Consequently, the intrinsic variability of S2 presents greater challenges for accurate segmentation and recognition of heart sound signals compared to S1. The precise segmentation by this algorithm not only encompasses recognition of basic heart sounds and murmurs but also accounts for high-density cardiac cycles in pediatric heart sound signals and variable durations of systolic and diastolic periods.

### 4.2. Classification

This study leveraged MFCC and its derivative features to delineate distinct patterns in heart sound signals between normal, ASD, and VSD conditions. The persistent split or accentuation of the second heart sound observed in ASD patients was characterized by specific spectral patterns in MFCC features. Conversely, the coarse holosystolic murmurs characteristic of VSD were represented by distinct low-frequency energy distributions. These featured differentiations established a basis for the automated classification of heart sounds. Utilizing a random forest classifier, we demonstrated that MFCC features and its derivatives could effectively discriminate among heart sounds across different conditions. One reason for the exceptional classification performance stems from the comprehensive exploitation of acoustic static and dynamics information [31], and another reason is the number of cycles of heart sound fragments. By applying principal component analysis, we reduced the extracted features to retain the most representative information while minimizing features’ spatial dimension, thus eliminating redundancy and noise in the signal for improved classifier accuracy and efficiency.

Accurate classification of heart sound signals is vital for the early diagnosis of heart disease. Nevertheless, the algorithm faces a challenge in distinguishing between ASD and VSD due to similarities in heart sound performance. For example, both conditions may lead to an enhanced second heart sound, potentially confusing the algorithm. Additionally, complications such as tricuspid regurgitation in patients with ASD and VSD further complicate the classification task.

### 4.3. Limitation

The segmentation algorithm has effectively achieved precise localization and segmentation of the cardiac cycle. Nevertheless, the algorithm’s performance may be compromised by significant noise interference or unclear boundaries within the heart sound signal, leading to positional errors in identifying the fundamental heart sound. Furthermore, the establishment of a tolerance time window based on the general durations of S1 and S2 across the dataset introduced potential bias in predicting the positions of S1 or S2. The number of cardiac cycles in the heart sound signal significantly influenced the performance of the random forest classifier. While a higher count of cardiac cycles yields richer feature information for effective classification decisions, it also introduced increased data processing complexity, impacting computing resources and time. Therefore, it is essential to strike a balance between enhancing classification performance and maintaining computational efficiency in practical applications. Potential avenues for future research encompass the expansion of sample sizes, the investigation of methods for sample enhancement, and the optimization of classification models, which are the primary directions our research is currently exploring and intends to develop further.

## 5. Conclusions

In this study, we successfully implemented a localization segmentation algorithm tailored for pediatric heart sound signals and conducted multi-type classification for congenital heart disease. The proposed algorithm efficiently extracts the envelope of the heart sound signal and utilizes the TBLSTM model to accurately locate each state of the heart sound signal, subsequently dividing the signal into fragments containing varying numbers of cardiac cycles. By extracting the MFCC and its derivative features in these fragments, combined with the random forest classifier, we achieved effective multi-classification of congenital heart disease. These results significantly enhance the automation level of heart sound signal analysis and offer a new technical approach for early diagnosis and classification of congenital heart disease.

## Figures and Tables

**Table 1:** Segmentation performance based on different methods.

Method	Acc	P	Se	$F_{1}^{S1}$	$F_{1}^{S2}$	$F_{1}$
LR-HSMM	92.12%	92.36%	92.18%	88.66%	81.14%	92.27%
CLSTM	92.74%	96.45%	85.65%	87.57%	82.61%	91.07%
FPN	87.07%	87.47%	86.73%	84.97%	78.14%	87.11%
TLSTM (proposed)	91.19%	91.45%	90.96%	86.41%	81.89%	91.21%
TBLSTM (proposed)	94.15%	94.21%	94.09%	90.25%	86.04%	94.15%

**Table 2:** Multi-classification performance of heart sound signals under different cardiac cycles.

Cycle	Acc	Se	$F_{1}$	$F_{1}^{ASD}$	$F_{1}^{VSD}$
1	72.03%	72.62%	72.32%	67.22%	76.95%
2	80.67%	80.90%	80.79%	78.08%	83.79%
3	85.07%	85.31%	85.19%	82.95%	87.35%
4	88.37%	88.79%	88.58%	86.76%	89.10%
5	90.57%	90.78%	90.67%	88.84%	91.70%
6	94.43%	94.58%	94.51%	93.24%	94.45%
7	94.77%	94.86%	94.81%	94.35%	94.25%
8	95.17%	95.26%	95.21%	94.32%	94.07%
9	95.97%	96.04%	96.00%	95.21%	94.89%

## Data Availability

The data presented in this article are available upon request from the corresponding author.
